# The training of wrist arthroscopy

**DOI:** 10.3389/fmed.2022.947459

**Published:** 2022-12-16

**Authors:** Haifei Shi, Pan Lu, Dongdong Yu, Jiwen Wang, Zhenhua Wang, Baotang Zhuang, Chao Shao, Chang Liu, Bo Liu

**Affiliations:** ^1^Department of Orthopedics, The First Affiliated Hospital, Zhejiang University School of Medicine, Hangzhou, Zhejiang, China; ^2^China Academy of Space Technology, Beijing Institute of Control Engineering, Beijing, China; ^3^Department of Hand Surgery, Beijing Ji Shui Tan Hospital, Xicheng District, Beijing, China

**Keywords:** wrist, arthroscopy, practitioner, training, education

## Abstract

The wrist is a complex joint that bridges the hand to the forearm. Patients with wrist disorders increasingly prefer minimally invasive procedures for wrist joint diagnosis and treatment. Wrist arthroscopy offers direct visualization of the structures of the joint anatomy and existing disease processes while causing minimal damage to surrounding soft tissue. However, it requires a high level of technical ability for wrist arthroscopy practitioners. Therefore, an improved focus on wrist arthroscopy training combining new educational media and traditional practice should aid in the development of novel wrist arthroscopy training mode. This article aims to describe the status of wrist training and evaluation systems and introduce a new progressive wrist training system.

## 1. Introduction

Increasing evidence confirms that wrist arthroscopy has unique advantages for the diagnosis and treatment of wrist injuries (1). For patients with wrist disorders, diagnosis purely based on medical history, physical examination, and radiological imaging has certain limitations. For complex wrist disorders, accurate assessment of the degree and type of wrist injury based on these modalities only may be impossible. Diagnostic arthroscopy, as a method of wrist joint examination under direct vision, forms the basis of accurate diagnosis of wrist-related diseases (2). For wrist-related diseases, surgical treatment based on wrist arthroscopy, compared with open surgery, has the advantages of smaller incisions, less surgical trauma, and less risk of joint damage, which can reach or exceed the curative effect of open surgery for some diseases (3). Currently, wrist arthroscopy is widely used in the diagnosis and treatment of triangular fibrocartilage complex injuries, interosseous ligament injuries, intra-articular fractures of the distal radius, and scaphoid fractures (4).

In recent years, patients with wrist disorders increasingly prefer minimally invasive procedures for wrist joint diagnosis and treatment. Therefore, the demand for wrist arthroscopy is gradually increasing. However, the wrist joint is complex because of its narrowness and the many bony and ligamentous structures. It is composed of multiple joint surfaces and ligaments, and its motion is multi-directional and multi-axial. Therefore, due to the steep learning curve, inappropriate surgery may increase the incidence of complications and affect the curative effect (2). In addition, compared with open surgery, arthroscopy requires specific surgical techniques (5), which require surgeons to perceive a three-dimensional environment from two-dimensional images and complete the surgeries through specific hand-eye coordination only with less tactile feedback. Therefore, a high level of technical ability is a necessity for wrist arthroscopy practitioners.

Analysis of the professional backgrounds of wrist arthroscopy practitioners—including arthroscopic, hand, and plastic surgeons—reveals that these professionals often do not possess both hand surgery and arthroscopic surgery skills. Although hand surgery is a mixed specialty between plastic and orthopedics, surgeons from the orthopedic specialty are more often called upon to perform arthroscopies on other joints and therefore have this “3D vision” more easily (6). Currently, most arthroscopy training programs adopt the progressive training method combined with basic knowledge teaching, stimulator training, cadaver course, and clinical practice. These programs have achieved good results. However, compared with other fields of arthroscopy training, such as knee and hip arthroscopy, wrist arthroscopy still lacks effective and reliable training programs. Contrary to the other joints, there is not yet a validated simulator for wrist arthroscopy. Currently, the cadaveric course is the only method for training in wrist arthroscopy (7). The disadvantages of cadaveric training are high costs, ethical issues, contamination risks, and limited availability of specimens. These disadvantages extensively limit wrist arthroscopy training. Therefore, a new wrist arthroscopy training model is needed to improve the quality of training and field of application.

This study synthesizes the current research status, reviews the status of wrist training and evaluation systems, and proposes a new progressive wrist training system.

## 2. The characteristics and difficulties of wrist arthroscopy training

The wrist joint has the following characteristics due to its complex anatomy and small operating space:

### 2.1. The complex anatomy of the wrist joint and diversity of its related diseases

The wrist joint is a complex entity, which is composed of multiple joints, including radial-carpal joint, intercarpal joints, and carpometacarpal joints. These joints are interrelated and collectively referred to as the wrist joint (8, 9). The complex articular and ligamentous anatomy contributes to the stability and function of the wrist joint (10). Fracture, dislocation, and ligament injuries of any of these joints can impair the structure and function of the wrist joint and result in a pathology (8, 9, 11). In wrist arthroscopy and treatment, the location of the portals is a special challenge for surgeons. A thorough knowledge of wrist anatomy is important to avoid possible complications. The location of wrist arthroscopy portals is diverse and complex; therefore, the location, structure, and nature of the injury should be considered to avoid neurovascular injuries (12). Appropriate portal placement is important for the success of diagnosis and treatment because of the reduced space, especially in arthritic or traumatic contexts, the perfect realization of the approach is necessary because the deformations leave much less space than inside a knee or a shoulder.

### 2.2. Operative skills of wrist arthroscopy

Conventional arthroscopy operation poses challenges, such as the need for profound knowledge of the anatomy, perception of a three-dimensional environment from a two-dimensional camera image, hand–eye coordination, and fulcrum effect (13). Due to the narrow internal structure and operating space of the joint, wrist arthroscopy uses alternative surgical instruments and methods rather than conventional large-joint arthroscopy. The 1.9-mm NanoScope with a 2.2-mm outer diameter cannula specifically designed for small joint visualization. It is lightweight and flexible, permitting access to the smallest of joints such as the DRUJ, MCPJ, and proximal interphalangeal joint (14). Wrist arthroscopy techniques combine the features of endoscopy and hand surgery, and surgeons need a unique set of surgical abilities to use such techniques.

### 2.3. The different and complex backgrounds of physicians in wrist arthroscopy

#### 2.3.1. Training

Currently, medical personnel involved in wrist arthroscopy training programs include plastic, hand, and joint surgeons. Based on their professional background, they were categorized into three groups: doctors familiar with the wrist's anatomy (hand surgeons); doctors familiar with arthroscopic surgery (joint surgeons); and doctors familiar with precision surgery (hand and plastic surgeons). These individuals with differing backgrounds need different training modes. For example, hand surgeons need emphasis on arthroscopic surgical skills and characteristics of anatomical structure from the perspective of arthroscopy. For joint surgeons, knowledge of wrist anatomy should be emphasized. In a study, it observed that residents have difficulty accessing wrist arthroscopy surgery during their training. 77.54% of them have seen between 0 and 10 wrist arthroscopies and 35.71% have never seen a wrist arthroscopy. In addition, only 23.80% of them have had access to training on cadavers, and 57.14% of them were trained in private facilities (15). Teaching students in accordance with their aptitude is particularly important in wrist arthroscopy training and new training methods could be adapted for wrist arthroscopy.

## 3. New forms of wrist arthroscopy training programs

### 3.1. E-learning courses

E-learning is an educational tool that uses computers, mobile phones, and other electronic devices as carriers and the internet as the medium to impart basic and advanced knowledge through interactive learning (16). According to Ruiz (17), E-learning refers to the use of internet technology to provide a wide range of solutions to improve knowledge and performance. Several studies have discussed the application of e-learning in medical education (18).

The development of computer technology has progressed e-learning. The advantage of e-learning lies in the flexibility of access to learning materials and regular evaluation. Currently, most physicians or hospitals are equipped with electronic devices that can be connected to the internet, which allows e-learning participants to freely choose the place and time of learning, and use fragmented time to learn. In addition, regular network tests or evaluations can assess the level of skill mastery of the students to select appropriate personalized training courses through big data processing to help each student progress.

One disadvantage of e-learning is that it does not provide contact between teachers and students; therefore, it should not completely replace in-person education (19). The limitations of building specific modules of e-learning affect the outcome of education. For example, Levinson et al. (20) showed that for learners with relatively low spatial ability, a multi-view modular approach to learn three-dimensional brain anatomy may be disadvantageous. In addition, the interactive control of e-learning content may adversely affect education (21). Behavioral activities, such are different from cognitive activities. E-learning may cause cognitive activities to be stimulated preferentially by passive and program-controlled learning material presentation methods rather than actual cognitive learning stimuli.

E-learning is especially useful in teaching theoretical knowledge. In a survey, Stevens (22) revealed that 90% of plastic surgery residents want to include online learning in their courses. Moreover, most reviews have reported the advantages of e-learning (21, 23–25). Some studies have explored the comparison of knowledge improvement between e-learning and traditional learning. Bhatti et al. (26) reviewed the effectiveness of e-learning by comparing traditional teaching methods and e-learning. The knowledge acceptance of the e-learning group was significantly higher than that of the traditional learning group. However, a study has shown that no significant difference in knowledge improvement exists between the e-learning group and traditional learning group (27). Additionally, Bhatti et al. (26) observed the satisfaction of students under the two education modes and found no significant difference between both groups.

E-learning can be a viable method in wrist arthroscopy training. Complex anatomical structures and novel surgical schemes can be effectively taught on e-learning platforms to promote quick and effective student mastery. Obdeijn et al. (28) developed a computer-based learning module for wrist arthroscopy. The learning enhancement effect of the module was tested in a randomized controlled trial (RCT). Twenty-eight medical students were assigned to the computer-based module group or lecture group. Although the computer-based module did not improve learning, learners found it more enjoyable to use. In the new Wrist Basecamp project of Pr C. Mathoulin, there are very few platforms in wrist arthroscopy and that these have training credit validations, which could potentially encourage surgeons to train or discover a wrist surgery culture. Because e-learning is increasingly becoming an important form of learning, further studies are needed to assess its rationality and effectiveness.

### 3.2. Simulator course

Surgical simulators can be divided into three types: box simulator, virtual reality (VR) simulator, and augmented reality simulator. The box trainer is a simple device. The trainee must observe the operative action on the screen and perform the operative task with the real instrument in the box (29–31). The box can be a simple square box or a physical model similar to the human body.

VR trainer is a computer-based application that allows you to move in free space, while performing tasks in a virtual operating environment on the computer screen. VR and physical models are often called augmented reality trainer after “superposition”. These types of simulators provide the advantages of both systems, such as tactile feedback, real surgical material use, physical contact with the model, realistic internal views, and training possibilities for different scenes. For arthroscopic technique training, several studies have fully investigated knee and shoulder arthroscopic simulators. They showed that training with an augmented simulator plays a significant role in the mastery of necessary arthroscopic skills. In 2008, Yaacoub (32) proposed the concept of wrist arthroscopic simulation. However, this system was not further developed. Some studies have shown that the use of VR simulation for training can reduce the time and number of errors in the execution of specific surgical tasks (33, 34). A multicenter clinical study confirmed that doctors who received VR training made fewer mistakes and performed better in the operating room (35). In addition, using RCTs, Cannon et al. (36) showed that the technical performance of joint surgery trainees trained using laboratory arthroscopic simulation for a period of time in the operating room was significantly better than that of the untrained group.

Currently, the apprenticeship training method is continually challenged by the tense relationship between doctors and patients. In clinical training with clinical patients, the operation time is prolonged, cost is increased, and operation risk of patients is elevated. These factors make the development of simulation particularly important. The large-scale cancellation of physical training programs during the COVID-19 pandemic highlights the importance of simulator training. Additionally, simulators are needed for better skill improvement; all surgeons need to learn from their mistakes, and this learning curve is best performed outside of clinical surgery. Simulation training provides a comfortable environment for students to make mistakes without dire consequences. In a simulation, effective feedback, repeated practice, and various learning strategies, in a personalized, controllable learning environment can promote training quality. Gallagher (37) defined attention ability threshold (ACT) as the amount of information of focus at a specific point in time. When psychomotor skills and spatial judgments become automatic while monitoring hand position or tool movement, the novice focuses more on treating the clinical problem.

Although simulations provide advantages of effective feedback, repetitive practice, multiple learning strategies, controlled learning environment, and individualized learning (32), they lack the characteristics of reality, and their tasks are not as complex as real tasks. The physical model simulating parts of the body or organ increases the reality of the simulator. In physical models, anatomical landmarks can be identified, and palpation and positioning are easier than in box trainers. The disadvantage is that such a model does not provide realistic simulation, such as bleeding, pain, and changes in vital signs (38, 39). However, the VR simulator can simulate the surgical complications concurrently. VR surgical simulators combine visual and tactile interfaces, which aim to help surgical students and residents to master complex surgical procedures. The advantage of VR system simulators is that they can monitor and evaluate performance objectively. However, the disadvantage is that they are generally expensive, which means that they may not be popularized on a large-scale (40, 41).

Currently, no suitable simulators are available for wrist arthroscopy training. Therefore, cadavers are still the most important teaching tools. Considering the complex structure of the wrist joint, the development of a simulator can be difficult. Seemingly, a simple large arthroscopic simulator can replace a part of the wrist arthroscopic training. Many specific arthroscopic techniques, such as triangulation (two-handed operation while observing the motion on the screen) and hand–eye coordination, can be implemented in the arthroscopic simulator. However, due to the particularity of the wrist joint anatomy, the large-joint arthroscopic simulator cannot completely replace the wrist arthroscopic simulators. A study has shown that doctors who have received knee arthroscopy training have no better training effect on wrist arthroscopic surgery (42). In addition, due to the limited field of vision and the limited physical interaction between doctors and patients, arthroscopic wrist surgery is more suitable for VR simulation than most other orthopedic surgeries.

The advantage of the simulator is not only in training but also in evaluating trainees' proficiency in surgery without supervision (43, 44). Robust data capture and real-time feedback of indicators make the assessment of students' skills concrete and objective. By installing the necessary programs on the VR arthroscopic simulator, an effective, reliable, and feasible test mode based on the basic arthroscopic operation ability can be created. Moreover, a reliable pass/fail standard can be established. Simulator-based testing and pass/fail criteria can help to assess and ensure the basic competence of future wrist arthroscopy practitioners before clinical practice (45).

According to a study on the demand of wrist arthroscopy-trained surgeons for e-learning and simulation (46), 55 out of 64 hand surgeons believed that wrist arthroscopy e-learning will become an important content of wrist arthroscopy training, whereas 60 out of 64 doctors believed that wrist arthroscopy simulation will be an important learning tool in the future. Although a wrist arthroscopy simulator is not yet available, cadaver courses and workshops remain effective (albeit expensive) training alternatives. Wrist arthroscopy is relatively safe, irreversible and patient-interfering injuries can occur, especially during the first training arthroscopy (47).

### 3.3. Cadaveric training

Cadaveric training is still the main training method for most joints, especially for small joint surgery. Cadaveric anatomical structures are similar to anatomical structures in clinical practice. Therefore, cadaveric training is the most appropriate training model for preclinical training and evaluation of trainees' professional skills. Currently, cadaveric training is mainly used in wrist training courses globally, forming a complete set of training programs for common wrist diseases. Puhaindran et al. (48) specifically performed cadaveric complications after a “first” arthroscopy. It observed high potential for injuries during wrist arthroscopy, especially when performed by novices. Thus, this study suggests that a trainee's first arthroscopies should be done on cadavers. Cadaveric training would increase their awareness of the potential iatrogenic injuries, and allow correction of any deficiencies in technique.

## 4. New training mode of wrist arthroscopy

Combined with our previous wrist arthroscopy training program, the curriculum design of wrist arthroscopy is still based on cadaveric teaching. The course, which is mainly delivered offline, includes surgical skills of wrist arthroscopy for existing diseases. This course design is effective for students with basic knowledge of wrist arthroscopy. However, for students with weak anatomical knowledge, endoscopic surgical skill training is necessary. Therefore, personalized courses and e-learning modes should be combined with offline learning for different students. Similarly, the course of cadaveric teaching aids can only be conducted on a small-scale and cannot be deployed at large-scale. Therefore, the appropriate potential simulator and virtual reality technology should be integrated into future courses to study their effectiveness.

## 5. Effect feedback on wrist arthroscopy training (evaluation system)

The improvement of training methods and curriculum design is inseparable from an effective feedback mechanism. Research on medical education, such as knowledge and skill acquisition, mostly considers effectiveness of the intervention as the main goal. Therefore, appropriate evaluation systems of knowledge acquisition and skill acquisition, and series of theoretical and operational evaluation systems, ranging from simple to complex systems, should be established. Furthermore, training satisfaction is an important feedback on the effect of training. Satisfaction should include many aspects, including learning content and methods (43).

In recent years, the study of objective evaluation methods for surgical skills has addressed ability-based training evaluation to a large extent to improve training content and programs (49). Common examples range from simple to complex: action analysis, specific task resolution, and global behavior evaluation. Motion analysis has been validated as an assessment tool for determining skill levels and can be performed in simulators or equipment with real-time recording. The task-based evaluation table is an evaluation system for specific surgical tasks. It is suitable for a single task, and it will show up as a ceiling effect in the early stage. The Global Rating Scale (GRS) serves as a global behavioral evaluation system to evaluate the performance of several skill areas to generate an overall performance rating. GRS has been used in simulators and clinical evaluations in multiple surgical specialties (50). The GRS used for arthroscopic surgery has proven to have a good ability to assess the subject's skill level and learning curve, as shown by excellent structural validity (the ability to detect differences between skill levels) and excellent performance reliability. Because no additional equipment or operating room is required and multiple tasks can be evaluated concurrently, GRS is a viable evaluation tool. For these commonly used arthroscopic GRS overall scoring scales, none of them shows significant advantages; therefore, any scoring system can be used. GRS generally includes a task list and scoring system (50). However, due to the particularity of wrist arthroscopy, the specific task table for GRS overall score scale for arthroscopy needs to be optimized to clarify the evaluation criteria for common surgical skills of wrist arthroscopy.

## 6. Clinical practice in different wrist disorders

Wrist arthroscopy allows the visualization of various wrist disorders, including injuries to the triangular fibrocartilage complex (TFCC), osteochondral lesion of the carpus, fractures, and carpal instability and radiocarpal arthritis. Clinical practice of wrist arthroscopy focuses on TFCC repairs, treatment of radiocarpal synovitis and arthritis, fracture reduction/fixation, non-union treatment, partial wrist joint fusion, and management of soft tissue pathologies such as ganglion excisions and release of contracture. Therefore, practitioners with different skill levels are provided with specified training courses to meet different stages of the learning curve (Table 1). Due to dramatical improvement of instrumentation and implants used in wrist arthroscopy, new surgical skills and arthroscopic techniques have continued to expand. Thus, modification of training program and surgical practice is required.

**Table 1:** Techniques in wrist and hand arthroscopy.

**Basic**	**Intermediate**	**Advanced**
•Radiocarpal and Midcarpal joint examination	•TFCC capsular repair	•TFCC tendon graft reconstruction
•Staging and debridement of avascular necrosis	•TFCC foveal repair	•SLIL tendon graft reconstruction
•Synovectomy and synovial biopsy	•Reduction and pinning of scapholunate instability	•Partial wrist joint fusion
•Loose body removal	•Treatment of interosseous ligament injuries: SLIL, LTIL	•Treatment of scaphoid fractures and non-unions
•Wrist Ganglionectomy	•Arthroscopic Wafer resection	•Treatment of distal radius fractures and intraarticular malunions
•Chondroplasty	•Bone resection: radial Styloidectomy, distal scaphoid resection, hamate pole resection	•STT joint arthroscopy with pyrocarbon interposition
•Staging and debridement of avascular necrosis		•Small Joint Arthroscopy: Thumb CMCJ, MCPJ, STT joint
•Release of wrist contracture		
•Carpal tunnel release		

TFCC, triangular fibrocartilage complex; SLIL, Scapholunate interosseous ligament; LTIL, Lunotriquetral interosseous ligament; STT, Scaphotrapeziotrapezoidal; CMCJ, Carpometacarpal joint; MCPJ, Metacarpophalangeal Joint.

## 7. Summary

The increasing popularity of wrist arthroscopy in the diagnosis and treatment of wrist disorders necessitates an improved focus on wrist arthroscopy training. Combining new educational media, such as e-learning and simulators, and traditional cadaveric practice and clinical experience, a progressive wrist arthroscopy training mode can be established. In reference to the arthroscopic training evaluation system, the application and improvement of action analysis, specific task solving, and global behavior evaluation can form an effective wrist arthroscopic training feedback system and improve training quality.

## Author contributions

All authors listed have made a substantial, direct, and intellectual contribution to the work and approved it for publication.
